# Coffee consumption and NAFLD: a community based study on 1223 subjects

**DOI:** 10.1186/s13104-015-1645-3

**Published:** 2015-11-03

**Authors:** Tilmann Graeter, Pia C. Niedermayer, Richard A. Mason, Suemeyra Oeztuerk, Mark M. Haenle, Wolfgang Koenig, Bernhard Otto Boehm, Wolfgang Kratzer, for the EMIL-Study group

**Affiliations:** Department of Diagnostic and Interventional Radiology, University Hospital Ulm, Albert-Einstein-Allee 23, 89081 Ulm, Germany; Department of Internal Medicine I, University Hospital Ulm, Albert-Einstein-Allee 23, 89081 Ulm, Germany; Louis Stokes Clevel and Department of Veterans Affairs Medical Center, 10700 East Boulevard, Cleveland, OH 44106 USA; Department of Internal Medicine II, University Hospital Ulm, Albert-Einstein-Allee 23, 89081 Ulm, Germany; LKC School of Medicine, Nanyang Technological University, Singapore, Singapore; Imperial College London, London, UK

**Keywords:** Caffeine, Hepatic steatosis, Fatty liver, Alanine aminotransferase, Population-based cross-sectional study

## Abstract

**Background:**

Objective of the present cross-sectional study was to investigate the impact of caffeine consumption on fatty liver and serum alanine aminotransferase (ALT) concentrations in a random population sample.

**Methods:**

All subjects (n = 1452; 789 women, 663 men; average age 42.3 ± 12.8 years) underwent ultrasonographic examination of the liver and completed a standardized questionnaire regarding personal and lifestyle data, in particular relating to coffee consumption and past medical history. In addition, anthropometric data were documented and laboratory examinations performed. Statistical interpretation of the data was performed descriptively and by means of bivariate and multivariate analysis.

**Results:**

Data of the present study demonstrated a significant association between hepatic steatosis male gender (p < 0.0001), advanced age (p < 0.0001) and elevated body-mass index (BMI; p < 0.0001). No association between caffeine consumption and fatty liver was identified. An association between caffeine consumption and elevated serum ALT concentrations was not identified.

**Conclusions:**

The findings of the present study provide no evidence for an association between caffeine consumption and either the prevalence of hepatic steatosis or serum ALT concentrations.

## Background

Non-alcoholic fatty liver disease (NAFLD) is the most frequent and most important liver disorder worldwide [1]. The increase in overweight, type-2 diabetes mellitus and dyslipidemia has reached epidemic proportions. The development of NAFLD is closely associated with all three of these disorders [2]. The spectrum of NAFLD extends from simple deposits of fat in the liver to non-alcoholic steatohepatitis (NASH), fibrosis and, ultimately, cirrhosis. Histology reveals triglyceride-containing fat vesicles within the hepatic parenchyma that are characteristic of simple fat deposits; NASH is characterized by inflammatory infiltrates and cell destruction [3]. In cirrhosis, scar tissue replaces the degenerated hepatic parenchyma.

Fatty liver is diagnosed using imaging methods such as ultrasonography (US), and magnetic resonance imaging (MRI). MRI is superior to ultrasound in detecting fatty liver, especially when the degree of hepatic steatosis is mild. Using ultrasound is practical and cost-effective and is associated with a sensitivity of 83 % and specifity of 100 % if at least 30 % of the liver has been affected by fat accumulation and has been shown to be very valuable clinically in assessing the fat content of the liver [4].

Laboratory parameters, such as an elevated transaminase quotient (aspartate transaminase (AST)/alanine transaminase (ALT)) or a depressed adiponectin concentration can provide some assistance in distinguishing steatohepatitis from simple fatty liver [5]. Liver biopsy with histological examination remains, however, the gold standard for diagnostic confirmation [6, 7].

Coffee is one of the most widely consumed beverages worldwide [8]. Beneficial effects of coffee consumption have been demonstrated for a wide variety of disorders, including Parkinson’s disease, diabetes mellitus, colorectal carcinoma, coronary artery disease, liver diseases and suicidality [9, 10]. Coffee consumption appears to even reduce mortality [9, 10].

Coffee consumption has its beneficial impact particularly on diseased or pathologically altered hepatic parenchyma. Several studies have shown that coffee consumption is associated with a less pronounced degree of fibrosis or cirrhosis of the liver [11–14], a lower bright liver score (BLS) [11, 15] and a lower prevalence and reduced severity of NAFLD [11, 16]. Furthermore, a number of studies have uncovered evidence that coffee consumption appears to associated with a lower concentration of ALT [17–19]. The influence of coffee consumption in hepatitis B patients remains controversial [20, 21].

To our knowledge, no studies of the effects of coffee consumption of hepatic steatosis and ALT concentrations in non-selected populations have been published. Objective of the present study was to investigate this potential relationship in a random population-based sample of individuals aged 18–65 years.

## Methods

### Study population/subjects

**Fig. 1:**
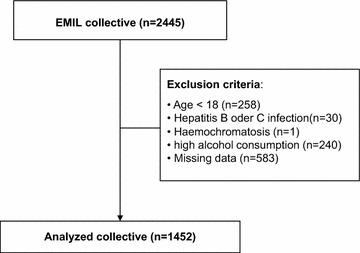
Flow of the subject across the study

The EMIL Study (**E**chinococcus **m**ultilocularis and other **I**nternal medical disorders in **L**eutkirch) was conducted in 2002 to investigate the respective prevalences of Echinococcus multilocularis infection and other disorders in the southwest German city of Leutkirch [22]. A total of 4000 registered inhabitants between the ages of 10 and 65 years received invitations by mail to participate in a random population-based sample. Of these, 2445 persons provided their written consent and were examined for the study. In order to determine the effects of coffee consumption on hepatic steatosis, certain exclusion criteria were established. Excluded from the study were all minors; all subjects with history of hepatitis B or C, or of hemochromatosis and all subjects reporting alcohol abuse (males, ≥40 g/d; females, ≥20 g/d). Also excluded were any subjects with incomplete data sets. The resulting study collective thus included 1452 subjects (Fig. 1).

### Quantification of caffeine consumption

Subjects’ consumption of caffeinated beverages such as coffee and black tea was assessed using a standardized questionnaire. Quantification of caffeine intake was based on the following categories: more than once a day, daily, less than weekly, less than monthly, seldom/rarely.

### Hepatic steatosis, ultrasonographic criteria

Four identical HDI 5000 units (ATL Ultrasound, Philips Medical Systems, Bothell, WA, USA) were used. The diagnosis of hepatic steatosis was made according to criteria proposed by Charatcharoenwitthaya based on a comparison of the hepatic and renal parenchyma, taking into consideration the dorsal attenuation, penetration of the diaphragm and ability to assess the liver vessels. The severity of the disease is defined as no steatosis and steatosis grade I, II and III [23, 24]. Due to the small number of cases in group III, these subjects were combined with those in group II for the statistical analysis.

### Laboratory tests and procedures

Laboratory blood testing included analytical clinical chemistry for liver enzymes, lipids and other biochemical values were determined using the Dimension XL unit (Dade Behring Inc., Newark, DE, USA).

### Metabolic syndrome: criteria

Metabolic syndrome was defined according to modified ‘US National Cholesterol Education Program Adult Treatment Panel III’ criteria, whereby at least three of the following criteria had to be met: Obesity with body-mass index (BMI) over 30 kg/m^2^; serum triglyceride (TG) concentration ≥1.69 mmol/l; serum high-density lipoprotein (HDL) cholesterol <1.29 mmol/l for women and <1.04 mmol/l for men; history of hypertension and random blood glucose >8.88 mmol/l or confirmed diabetes mellitus [24].

### Statistical analysis

The statistical analysis and calculations were performed with the SAS statistical software program (version 9.2). First, data were analyzed descriptively: for qualitative variables, the relative and absolute frequency were calculated, for quantitative variables the mean and standard deviation. In order to detect differences between subjects with and without hepatic steatosis, the Wilcoxon rank-sum test was used for continuous variables, while for categorical variables, the Chi-Square test were used. Univariate logistic regression was used to assess the association between caffeine consumption and hepatic steatosis. Multivariate logistic analysis was performed to evaluate the association between hepatic steatosis and caffeine consumption as well as other risk factors like age, BMI, gender, metabolic syndrome and physical activity. To assess the association between elevated ALT concentrations and coffee consumption univarite and multivariate logistic regression analysis was used.

All tests were two-tailed. Statistical significance was set at α = 0.05. The P value was given to four decimal places, while the odds ratio (OR) and 95 % confidence interval (CI) were given to three decimal places.

### Ethical agreement and informed consent

The study meets the criteria of the revised version of the World Medical Association Declaration of Helsinki (2000) concerning ethical principles for medical research involving human subjects and was approved by the ethics committee of the State Medical Association of Baden-Württemberg. Each study participant provided his or her written consent.

## Results

### Study subject demographics

**Fig. 2:**
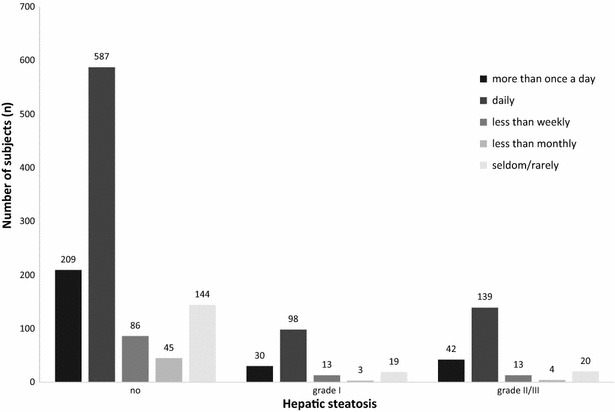
Caffeine consumption in relation to the severity of hepatic steatosis

The data of 1452 subjects [789 women (54.3 %), 663 men (45.7 %), average age 42.3 ± 12.8 years (range 18–65 years)] were analyzed in the present study. The majority of the subjects were of German nationality (89.9 %). The mean waist-to-hip ratio (WHR) stood at 0.8 ± 0.1, the mean BMI was 25.6 ± 4.7 kg/m^2^. Criteria for metabolic syndrome were met in 5.9 % of subjects. Hepatic steatosis was diagnosed in 381 subjects (26.2 %) and is more common in men (34.8 %, n = 231) than in women (19.0 %, n = 150). The difference in the frequency of hepatic steatosis between men and women was statistically significant (p < 0.0001). Grade I steatosis was identified in 163 subjects (11.2 %), and grade II/III steatosis was identified in 218 subjects (15.0 %; Fig. 2). Subjects with hepatic steatosis had higher BMI, waist-to-hip ratio (WHR) and transaminase levels (ALT, AST, GGT; Table 1). Elevated ALT concentrations were identified in 198 (13.6 %) subjects.

**Table 1:** Demographics and other characteristics of subjects with and without hepatic steatosis

	Hepatic steatosis not diagnosed (n = 1071, mean ± STD)	Hepatic steatosis diagnosed (n = 381, mean ± STD)	Total	*p *value
Gender, n (%)				
Female	639 (59.7 %)	150 (39.4 %)	789 (54.3 %)	*<0.0001*
Male	432 (40.3 %)	231 (60.6 %)	663 (45.7 %)	
Age	39.9 ± 12.4	49.0 ± 11.4	42.3 ± 12.8	*<0.0001*
BMI	24.1 ± 3.8	29.8 ± 4.7	25.6 ± 4.7	*<0.0001*
Waist/hip-ratio	0.8 ± 0.1	0.9 ± 0.1	0.8 ± 0.1	*<0.0001*
Coffee consumption, n (%)				
More than once a day	209 (19.5 %)	72 (18.9 %)	281 (19.4 %)	
Daily	587 (54.8 %)	237 (62.2 %)	824 (56.8 %)	
Less than weekly	86 (8.0 %)	26 (6.8 %)	112 (7.7 %)	
Less than monthly	45 (4.2 %)	7 (1.8 %)	52 (3.6 %)	*0.0408*
Seldom/rarely	144 (13.5 %)	39 (10.2 %)	183 (12.6 %)	
ALT	13.3 ± 5.6	20.0 ± 10.6	15.1 ± 7.8	*<0.0001*
AST	8.9 ± 2.4	11.0 ± 5.1	9.5 ± 3.5	*<0.0001*
GGT	11.0 ± 11.0	19.1 ± 20.3	13.2 ± 14.5	*<0.0001*
Metabolic syndrome, n (%)				
No	1050 (98.0 %)	317 (83.2 %)	1367 (94.2 %)	*<0.0001*
Yes	21 (2.0 %)	64 (16.8 %)	85 (5.9 %)	

Significant *p* values are in italics

*STD* standard deviation, *BMI* body-mass index, *ALT* alanine transaminase, *AST* aspartate transaminase, *GGT* gamma-glutamyl transferase

In all, 76.2 % of subjects consumed caffeine at least daily. In our study collective, there was no statistically significant difference between the sexes in terms of caffeine consumption (p = 0.1748). In the univariate analysis, a significant association could be identified between caffeine consumption and hepatic steatosis (p = 0.0461) .

Multivariate logistic analysis was performed to evaluate the association between hepatic steatosis and caffeine consumption as well as other risk factors like age, BMI, gender, metabolic syndrome and physical activity. The results show a significant association of heaptic steatosis with gender, age and BMI (p < 0.0001). A relationship between hepatic steatosis and caffeine consumption could not be shown (p = 0.8144, Table 2).

**Table 2:** Logistic regression analysis to assess the association between hepatic steatosis and caffee consumption and other influencing factors

	Odds ratio (95 % CI)	*p* value
Gender		*<0.0001*
Female	Ref.	
Male	2.775 (2.047–3.764)	
Age	1.053 (1.039–1.066)	*<0.0001*
BMI	1.324 (1.271–1.380)	*<0.0001*
Metabolic syndrome		
No	Ref.	Ref.
Yes	0.906 (0.501–1.640)	0.0668
Coffee consumption		
More than once a day	0.771 (0.443-1.343)	
Daily	0.809 (0.496-1.318)	
Less than weekly	0.749 (0.368–1.526)	0.8144
Less than monthly	0.558 (0.190–1.638)	
Seldom/rarely	Ref.	
Physical activity		
No	Ref.	
0–2 h/week	1.021 (0.719–1.449)	0.9187
2–4 h/week	0.931 (0.612–1.419)	
4–10 h/week	0.937 (0.548–1.603)	
>10 h/week	0.581 (0.169–2.001)	

Significant *p* values are in italics

*CI* confidence interval, *Ref.* reference group

In a further analysis, the impact of coffee on ALT concentrations was assessed. Both in the univariate analysis and after adjusting for gender, age and BMI, there is no significant association between coffee consumption and elevated ALT concentrations (p = 0.7495; p = 0.7905; Table 3).

**Table 3:** Regression analysis to assess the association between coffee consumption and elevated alanine transaminase levels (ALT)

Coffee consumption	Odds ratio (95 % CI)	p value
**Univariate**		
More than once a day	1.097 (0.617–1.953)	
Daily	1.302 (0.794–2.135)	
Less than weekly	1.380 (0.694–2.746)	0.7495
Less than monthly	1.006 (0.384–2.640)	
Seldom/rarely	Ref.	
**After adjusting for age, gender and BMI**		
More than once a day	0.773 (0.447–1.339)	
Daily	0.806 (0.497–1.308)	0.7905
Less than weekly	0.722 (0.355–1.467)	
Less than monthly	0.554 (0.190–1.613)	
Seldom/rarely	Ref.	

*CI* confidence interval, *Ref.* reference group

## Discussion

To date to our knowledge, no prospective, sonographic studies of the association between coffee consumption and NAFLD in large population-based collectives have been published. Previous community-based studies have investigated collectives with small numbers of subjects [13, 15] or have been retrospective and did not employ diagnostic ultrasonography [16]. Birerdinc et al. retrospectively analyzed 18,550 datasets from the NHANES study. The diagnosis of NAFLD, however, was made exclusively on the basis of elevated transaminase concentrations. Subjects in the NAFLD group consumed significantly less coffee than did the control group (p = 0.0003) [16]. Studies of elevated ALT concentrations showed significant differences with respect to the populations studied and do not permit any definitive conclusions regarding NAFLD as a cause of the ALT elevation [25]. The findings of this study, therefore, cannot be compared with our findings. Molloy et al. examined a patient collective using both ultrasonography and biopsy, and found that increased coffee consumption was associated with a reduced risk of liver fibrosis in patients with NASH [13]. For ethical reasons, histological diagnosis of NASH cannot be justified in a population-based study; hence, these data also cannot be directly compared with our own.

In a case–control study of a collective of 130 individuals (with and without hepatic steatosis in n = 73 and n = 57 subjects, respectively), Gutiérrez-Grobe et al. made the diagnosis of NAFLD solely on the basis of ultrasonographic criteria. As in our study, the severity of hepatic steatosis was divided into four grades. The findings of the study failed to demonstrate an unequivocal anti-oxidant effect of coffee though patients with higher-grade steatosis drank more coffee than did those with less severe steatosis [26].

In a study of 157 patients and 153 controls, Catalano et al. made the diagnosis of hepatic steatosis using different exclusion criteria. Coffee consumption was monitored over a follow-up period of 6 months. The authors thus ascribe a possible protective effect to elevated coffee consumption. Their data, however, were derived from a highly selected collective drawn from ambulatory patients of a gastroenterological practice and outpatient clinic and cannot be applied to the general population [15].

In our study population, elevated ALT concentrations were identified in 13.6 % of subjects. This corresponds with the finding of another cross-sectional study from China (10.8 %) [25]. Studies published to date have shown an inverse relationship between coffee consumption and serum ALT concentrations. The findings of our population-based study failed to demonstrate a corresponding association between caffeine consumption and serum ALT concentrations. It is to be noted that, in the other studies, the diagnosis of NAFLD was not based on imaging of the liver; furthermore, some of these studies were performed in highly selected populations [19, 27].

The present study does have some important limitations. Due to the choice of study design, we were able to investigate associations but not causalities. The diagnosis of hepatic steatosis was made on the basis of ultrasonographic criteria. Liver biopsy, the current gold standard for confirming a diagnosis of NAFLD, was not performed. Furthermore, data on caffeine consumption were obtained only on one occasion. In addition, the dual effect of coffee consumption was not investigated further.
